# Supplementary material for: The Association between Plasma Omega-6/Omega-3 Ratio and Anthropometric Traits Differs by Racial/Ethnic Groups and NFKB1 Genotypes in Healthy Young Adults
Source: J Pers Med. 2019 Feb 16;9(1):13. doi: 10.3390/jpm9010013 (PMC6462983; doi:10.3390/jpm9010013)
Supplement: Supplementary file 1 [file jpm-09-00013-s001.pdf]

Supplementary Table 1. Description of the polymorphisms in the *NFKB1* gene.

dbSNP	Position	Reference SNP Allele	MAF	Genotype frequency Homozygous major n (%)	Heterozygous n (%)	Homozygous minor n (%)
rs11722146	Intron	A/G	A	GG	GA	AA
			0.34	392 (43.7)	394 (43.9)	112 (12.5)
rs13117745	Intron	C/T	T	CC	CT	TT
			0.12	701 (78.1)	177 (19.7)	20 (2.2)
rs1609798	Intron	C/T	T	CC	CT	TT
			0.25	402 (44.8)	377 (42.0)	119 (13.3)
rs4648022	Intron	C/T	T	CC	CT	TT
			0.05	811 (90.3)	81 (9.0)	6 (0.7)
rs4648090	Intron	A/G	A	GG	GA	AA
			0.07	775 (86.3)	114 (12.7)	9 (1.0)
rs1599961	Intron	A/G	A	GG	GA	AA
			0.38	361 (40.2)	398 (44.3)	139 (15.5)
rs230511	Intron	A/G	A	GG	GA	AA
			0.36	379 (42.2)	396 (44.1)	123 (13.7)
rs7674640	Intergenic	C/T	C	CC	CT	TT
			0.49	230 (25.6)	450 (50.1)	218 (24.3)
rs3774932	Intron	A/G	G	AA	AG	GG
			0.50	223 (24.8)	454 (50.6)	221 (24.6)

Position and reference SNP alleles are from dbSNP short genetics variations NCBI reference assembly. Abbreviations: MAF, minor allele frequency; SNP, single nucleotide polymorphism.

Supplementary Table 2. *NFKB1* genotype frequency by racial/ethnic groups.

dbSNP	Racial / ethnic group	Genotype frequency Homozygous major n (%)	Heterozygous n (%)	Homozygous minor n (%)	P
rs11722146		GG	GA	AA	<0.0001
	C	216 (24.1)	192 (21.4)	47 (5.2)	
	EA	115 (12.8)	167 (18.6)	56 (6.2)	
	SA	60 (6.7)	35 (3.9)	9 (1.0)	
rs13117745		CC	CT	TT	<0.0001
	C	335 (37.4)	106 (11.8)	14 (1.6)	
	EA	298 (33.2)	38 (4.2)	2 (0.2)	
	SA	67 (7.5)	33 (3.7)	4 (0.5)	
rs1609798		CC	CT	TT	0.0009
	C	219 (24.4)	187 (20.9)	49 (5.5)	
	EA	124 (13.8)	156 (17.4)	58 (6.5)	
	SA	58 (6.5)	34 (3.8)	12 (1.3)	

rs4648022		CC	CT	TT	<0.0001
	C	382 (42.6)	67 (7.5)	6 (0.7)	
	EA	338 (37.7)	0 (0)	0 (0)	
	SA	90 (10.0)	14 (1.6)	0 (0)	
rs4648090		GG	GA	AA	<0.0001
	C	349 (38.9)	97 (10.8)	9 (1.0)	
	EA	334 (37.2)	4 (0.5)	0 (0)	
	SA	91 (10.1)	13 (1.5)	0 (0)	
rs1599961		GG	GA	AA	0.03
	C	189 (21.1)	195 (21.7)	71 (7.9)	
	EA	117 (13.0)	165 (18.4)	56 (6.2)	
	SA	54 (6.0)	38 (4.2)	12 (1.3)	
rs230511		GG	GA	AA	<0.0001
	C	209 (23.3)	191 (21.9)	55 (6.1)	
	EA	108 (12.0)	170 (19.0)	60 (6.7)	
	SA	61 (6.8)	35 (3.9)	8 (0.9)	
rs7674640		CC	CT	TT	0.48
	C	114 (12.7)	236 (26.3)	105 (11 7)	
	EA	81 (9.0)	165 (18.4)	92 (10.3)	
	SA	23 (2.6)	49 (5.5)	32 (3.6)	
rs3774932		AA	AG	GG	0.39
	C	123 (13.7)	229 (25.5)	103 (11.5)	
	EA	74 (8.3)	171 (19.1)	93 (10.4)	
	SA	24 (2.7)	54 (6.0)	26 (2.9)	

P values were estimated using a Chi-squared test. Abbreviations: C, Caucasians; EA, East Asians; SA, South Asians.
